# Research on the performance of MXMCCC materials for gas leakage sealing

**DOI:** 10.1038/s41598-025-94048-4

**Published:** 2025-03-20

**Authors:** Mingming Fu, Mengyao Wang, Xiaowei Qin, Weikang Cao, Lu Chen, Haotong Zhao, Yuping Zhang, Dongying Lang, Shun Liu, Xueqing Qin

**Affiliations:** 1 College of Civil Engineering and Architecture, Shandong University of Aeronautics, Binzhou, 256600 Shandong China; 2 Shanxi Jingu Coal Industry Co., Ltd, Linfen, 041000 Shanxi China; 3 Binzhou Construction Engineering Construction Drawing Review Center, Binzhou, 256600 Shandong China

**Keywords:** Gas leakage prevention, Multi-walled carbon nanotubes, Microstructure, Mechanical properties, Civil engineering, Materials science

## Abstract

To address the issues of poor strength and low efficiency in traditional clay-cement composite gas-sealing materials (CCC), a method was proposed to prepare a new type of sealing material by utilizing multi-walled carbon nanotubes (MWCNTs) along with xanthan gum (XG) and magnesium oxide (MgO) to modify CCC. Through controlled experiments of water extraction rate testing, the optimal water-to-solid ratio for the multi-component system material has been determined to be 0.6. Mechanical performance testing reveals that when 1.5% xanthan gum, 5% magnesium oxide, and 1.39% multi-walled carbon nanotubes are added, the compressive strength of the multi-walled carbon nanotube-xanthan gum-magnesium oxide-clay-cement composite (MXM-CCC) reaches 18.60 MPa, with a flexural strength of 3.89 MPa. Pore integration analysis reveals that MXM-CCC has a porosity of 17.29%, with pore sizes ranging from 2.00 nm to 50 nm accounting for 71.46% of the total. The proportion of larger pores has decreased, resulting in a more optimal distribution of pore sizes. The formation mechanism and sealing mechanism of MXM-CCC were explored using characterization techniques such as XRD, FTIR, SEM, and thermogravimetric analysis. The hydroxyl and carboxyl groups in konjac gum undergo chelation with Ca²⁺ in CCC, forming a chelate structure. This causes the hydration products of the clay and cement to adhere together, improving the pore structure and mechanical properties of MXM-CCC. The addition of multi-walled carbon nanotubes accelerates the hydration reaction, increasing the content of substances such as C-(A)-S-H gel, ettringite, and Mg(OH)_2_ in the MXM-CCC. These chemicals act as a framework, providing support within the pores and inhibiting the shrinkage of MXM-CCC, and improving the adhesion between various hydration products. Additionally, multi-walled carbon nanotubes perform a nano-filling role, filling the pores and improving the density of the multi-component material, thereby enhancing its mechanical properties.

## Introduction

In 2023, China produced 47.1 billion tonnes of raw coal^1^. The expanded mining of coal has led to an increasing area of mined-out regions, a rise in the number of ventilation channels, and a larger probability of spontaneous combustion, creating safety hazards for underground workers and production equipment^2^. Therefore, preventing the spontaneous combustion of coal is essential to underground safety work^3^.

Fires caused by spontaneous combustion of coal account for over 90% of all mine fires, and the primary means of controlling these fires is to reduce or eliminate the supply of oxygen. Sealing gas leaks is a critical technology in preventing coal spontaneous combustion^4,5^. The spray sealing technology for gas leakage prevention is widely used in mining gob areas due to its convenient material sourcing, simple production process, lower cost, and flexibility^6,7^. Currently, the commonly used spraying materials of gas sealing in coal mines consist of organic and inorganic materials. The organic spraying materials of gas sealing primarily include phenolic foam, urea-formaldehyde foam, and polyurethane foam^8^. Yu et al.^9^ used phenol, urea, and paraformaldehyde as raw materials, curing agent, surfactant, and foaming agent as additives, and polyvinyl alcohol (PVA) and cashew nut shell oil as toughening agents into the resin to produce a novel polyurethane foam (PUF). This foam exhibited several advantages in terms of expansion performance, dimensional stability, compressive strength, and construction safety. Xi et al.^10^ studied the surfactants’ effects on the performance of composite foam slurry for mine sealing (CFSM). The foam gel sealing material developed can effectively prevent the spontaneous combustion of coal. Dong et al.^11^ found that urethane-modified phenolic foam (PF) could effectively reduce CO concentration and provide good sealing for gas leaks. Organic spraying materials of gas sealing are characterized by simple application, high expansion rates, good adhesion, and effective sealing. However, due to the flammable nature of some varieties and cost limitations, their more comprehensive application is challenging.

Inorganic spraying materials of gas sealing mainly include various modified cement mortar and composite slurry spraying materials of gas sealing. Xi et al.^12^ developed a new form of cement-based foam material (CBFM) with good sealing properties and impact resistance by selecting different types of surfactants and organic polymer (OP). Xue et al.^13^ synthesized a Janus-type composite solidify foam (JCSF) using non-combustible inorganic materials such as cement and fly ash as the matrix, with organic materials serving as the reinforcing component. The foam exhibited high sealing efficiency and excellent fire prevention and extinguishing performance. Li et al.^14^ used ordinary Portland cement as the base material and modified it with fly ash and other additives to develop a novel underground sealing material that had good rheological properties. Zhang et al.^15^ have developed a new spray sealing material by mixing cement with fly ash, silica fume, sand, water, and polypropylene fiber as spraying additives in appropriate proportions. Han et al.^16^ analyzed the characteristics of modified cement mortar spraying materials of gas sealing, exploring the effects of powder additives, water-reducing agents, and quick-setting agents through experimentation. He et al.^17^ conducted a study on the microstructural characteristics of hydrated clay-cement slurry, discovering that a significant amount of clay-cement gel clusters and C-S-H gel formed during the hydration of the clay-cement slurry. The clay-cement gel clusters were adsorbed onto each other and linked together, while C-S-H filled the gaps and connected to form a network.

Researchers have found that clay-cement-based mortar spraying materials are inexpensive and offer relatively excellent performance. However, most modified cement mortar spraying materials of gas sealing have problems such as excessive additives, complex production processes, long setting times, easy detachment, shrinkage and cracking after setting, which are not conducive to sealing gas leakage channels^18,19^. Therefore, enhancing the mechanical properties and crack resistance of clay-cement-based materials is crucial for optimizing spray sealing materials. Li et al.^20^ found that Xanthan gum could serve as a dispersing agent for cement hydration products, increasing the resistance of sodium silicate to dynamic dispersion. Cao et al.^21^ used magnesium oxide as a concrete expansion agent to reduce the water demand for concrete. The new material was stable in expansion and met the expected assumptions. Zhang et al.^22^ proposed using xanthan gum an magnesium oxide modification to prepare a new clay-cement-based spray sealing material. The new material exhibited strong stability and reduced porosity. Shi et al.^23^ utilized multi-walled carbon nanotubes, which were incorporated into a cement matrix after ultrasonic dispersion for modification purposes. Liu et al.^24^ used nanoindentation techniques to analyze the mechanical properties of cement-based materials containing multi-walled carbon nanotubes at the nanoscale. Liu et al.^25^ found that incorporating 0.06% graphene resulted in a certain improvement in the compressive and flexural strengths of cement-based materials. Wang et al.^26^ found that incorporating carbon nanotubes helped reduce the total porosity within cement-based materials. Liao et al.^27^ employed a chemical co-deposition method to prepare magnesium oxide-coated carbon nanotube samples, concluding that there was good interfacial compatibility between the magnesium oxide nanoparticles and the carbon nanotubes.

In summary, existing organic solidified foams generally have low durability, crumbling tendency, and high cost, making them unsuitable for large-scale application. Although modified cement mortar materials are more suitable for mass application, they suffer from poor adhesion and low strength, and tend to crack, creating gas leakage channels. Xanthan gum (XG) and magnesium oxide (MgO) can improve the pore structure of the material, enhancing its toughness and strength. Multi-walled carbon nanotubes (MWCNTs) contribute to the mechanical properties of cement mortar and exhibit good interfacial compatibility with MgO. As a result, this study utilizes MWCNTs, along with MgO and XG, to modify clay-cement-based composites (CCC). Through controlled experiments, samples were prepared with varying mass fractions of MWCNTs. Various properties of the samples were analyzed, enabling the selection of a sample that exhibits excellent mechanical performance, high strength, and good durability as a novel spray sealing material, namely, multi-walled carbon nanotubes-xanthan gum-magnesium oxide-clay-cement composite sealing materials (MXM-CCC).

## Experimental materials and methods

### Experimental materials

Cement (PO42.5), clay (kaolin, natural moisture content of 3%), MgO (analytical grade, calcined), XG (11138-66-2, McLean), sodium silicate (analytical grade), defoamer (organic silicon defoamer) and MWCNTs (carboxylated, industrial grade) were used in this study. Properties of experimental materials are shown in Table 1.

**Table 1:** Basic physical and chemical properties of experimental materials.

Name of material	Main components	Physical and chemical properties
Cement	(3CaO·SiO_2_, C3S) 50–70% (2CaO·SiO_2_, C2S) 15–30% (3CaO·Al_2_O_3_, C3A) 5–10% (4CaO·Al_2_O_3_·Fe_2_O_3_, C4AF) 5–15%	Heat of hydration: 250–350 J/g (72 h) initial condensation ≥ 45 min, final condensation ≤ 600 min (GB 175–2007) Specific surface area: 300–350 m^2^/kg
Clay	Kaolin type (1:1 layered silicate) Impurities: quartz (SiO_2_), hematite (Fe_2_O_3_)	Kaolin 3–15 meq/100 g Montmorillonite 80–150 meq/100 g
Magnesium oxide	Lightly burned MgO grain size 20–50 nm Impurities: CaO < 0.5%, SiO_2_ < 0.3%	Light burning type 120–300 s, Scorch reduction: 5–10% for light burning type
Sodium silicate	Alkaline sodium water glass (M = 1.0–2.0) Impurities: Fe_2_O_3_ < 0.05%, Al_2_O_3_ < 0.1%	Viscosity-temperature characteristics: Arrhenius exponential variation (activation energy 15–25 kJ/mol)
Xanthan gum	β-1,4-glucose main chain + trisaccharide side chain (D-mannose-β-1,4-D-glucuronide-β-1,2-D-mannose) Acetyl content: 4.5–5.5% Pyruvic acid content: 1.5-3.0%	Zero shear viscosity: η₀ = 1–5 Pa·s (1% solution) Pseudoplasticity index (n): 0.2–0.4 Dynamic modulus: G’ > G’’ (1 Hz)
Multi walled carbon Nanotubes	Defect density: ID/IG = 0.8–1.5 Metal impurities: Fe < 3 wt% Surface functional group: -COOH 1–5%	L/D ratio: 100–1000 Carrier mobility: 10^4^-10^5^ cm^2^/(V·s) Thermal conductivity: 1500–3000 W/(m·K)

### Experimental methods

Referring to the literature [22] for the optimization of clay to cement ratio and water-solid ratio, several groups of CCC samples were prepared, and the water-solid ratios were selected as 0.6, 1.0, and 1.5, and the clay accounted for 0, 10%, 20%, 30%, and 40% of the mass of the cement, respectively, in order to determine the water-solid ratios and the clay to cement ratios. The specific ratios are shown in Table 2.

**Table 2:** Dosage of components for performance test of the slurry.

Serial Number	Quality (g)		Water-to-solid ratio
	Cement	Clay	
1	200	0	0.6
2	200	20	0.6
3	200	40	0.6
4	200	60	0.6
5	200	80	0.6
6	200	0	1.0
7	200	20	1.0
8	200	40	1.0
9	200	60	1.0
10	200	80	1.0
11	200	0	1.5
12	200	20	1.5
13	200	40	1.5
14	200	60	1.5
15	200	80	1.5

#### Water separation rate test of the slurry

**Fig. 1:**
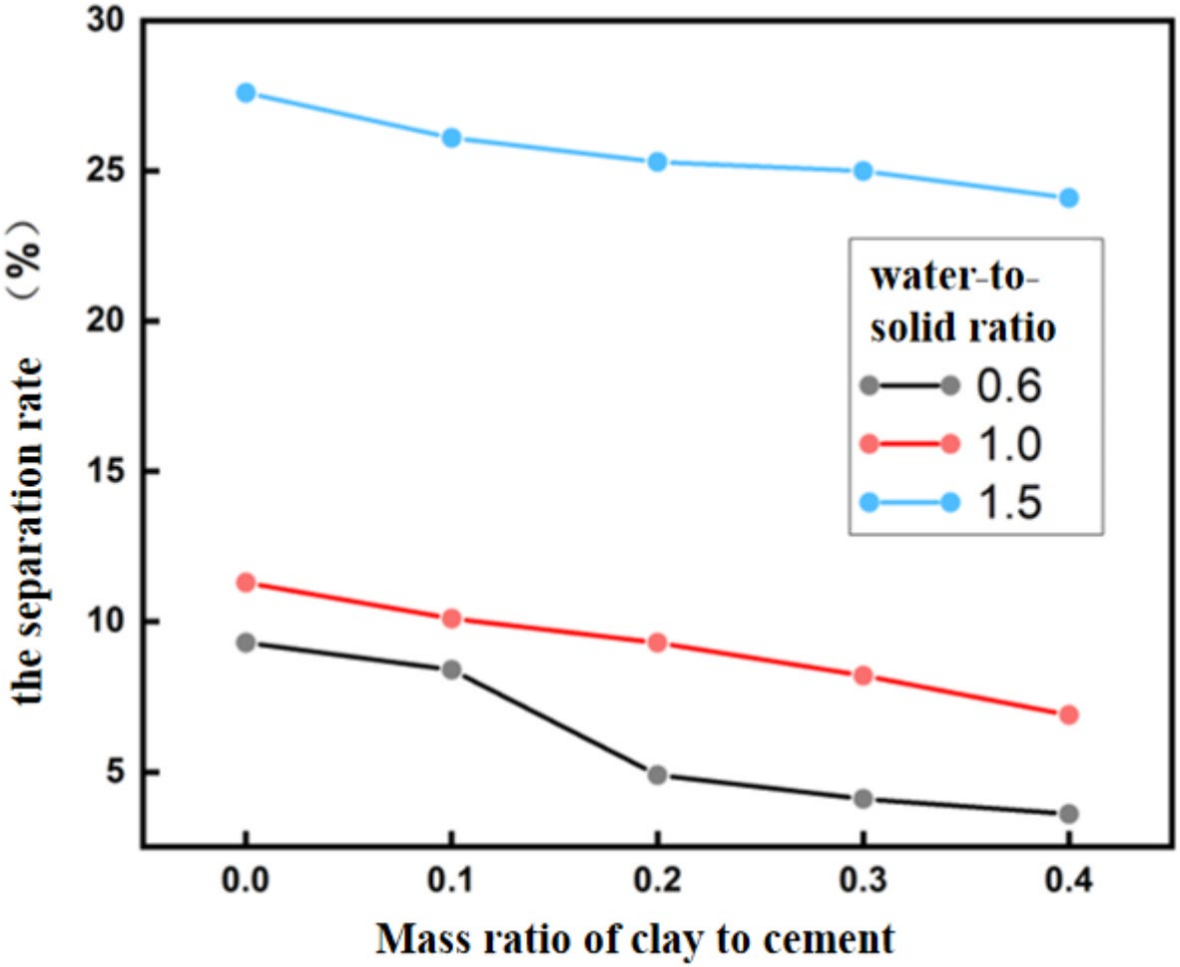
Water separation rate of CCC slurry after 2 h.

After mixing the slurry of clay and cement with water, it was poured into a 50mL test tube, and the initial height of the suspended liquid h0 was recorded. After 2 h, the height of the suspended liquid h1 was measured. The water separation rate of the slurry was calculated using Eq. (1)^28^. The water separation rate under each water-to-solid ratio are shown in Fig. 1.

1 $$\alpha = \frac{{h_{0} - h_{1} }}{{h_{0} }}$$

Figure 1 shows that as the mass ratio of clay to cement increases, the water separation rate from the slurry decreases under all water-to-solid ratios. However, it is evident that, at a constant mass ratio, increasing the water-to-solid ratio leads to a gradual rise in the water separation rate, which becomes significantly apparent when the water-to-solid ratio exceeds 1. When the solid-to-water ratio is 0.6, and the mass ratio of clay to cement is 0.2, the water separation rate of the slurry after 2 h can drop to below 5%.

On the basis of the experiments in Table 2, combined with the analysis of water precipitation rate, a water-solid ratio of 0.6 and a clay to cement ratio of 0.2 were selected. Referring to the literature^22^, the CCC system was supplemented with XG accounting for 0% (0 g), 0.5% (4.5 g), 1% (9 g), and 1.5% (13.5 g) of the mass of the cement, and with 0% (0 g), 2.5% (22.5 g), 5% (45 g), 7.5% (67.5 g) and 7.5% (67.5 g) of MgO, assisted by the addition of sodium silicate and defoamer for the experiment, in order to determine the optimum mixing ratio in the CCC system. The specific design is shown in Table 3.

**Table 3:** Dosage of components in the XG/MgO composite system.

Serial number	Quality (g)						Water-to-solid ratio	
	Cement	Clay	Sodium silicate	Defoamer	MgO	XG		
1	900	180	15	2	0	0	0.6	
2	900	180	15	2	0	4.5	0.6	
3	900	180	15	2	0	9	0.6	
4	900	180	15	2	0	13.5	0.6	
5	900	180	15	2	22.5	0	0.6	
6	900	180	15	2	22.5	4.5	0.6	
7	900	180	15	2	22.5	9	0.6	
8	900	180	15	2	22.5	13.5	0.6	
9	900	180	15	2	45	0	0.6	
10	900	180	15	2	45	4.5	0.6	
11	900	180	15	2	45	9	0.6	
12	900	180	15	2	45	13.5	0.6	
13	900	180	15	2	67.5	0	0.6	
14	900	180	15	2	67.5	4.5	0.6	
15	900	180	15	2	67.5	9	0.6	
16	900	180	15	2	67.5	13.5	0.6	

#### Mechanical performance analysis

According to *GB /T 17,671—1999*, after curing the specimens under standard conditions for 7 days, their mechanical properties were assessed using an electronic universal testing machine. Each group of specimens was tested three times, and the flexural and compressive strengths were calculated using Eqs. (2) and (3), respectively, and the average values were taken^29^.

2 $$R_{f} = \frac{{1.5F_{f} L}}{{b^{3} }}$$

3 $$R_{C} = \frac{{F_{C} }}{A}$$

In the formula, *R*_*f*_ represents the flexural strength, *F*_*f*_ denotes the flexural failure load, *L* indicates the distance between supporting cylinders, *b* refers to the edge length of the square cross-section of the prism, *R*_*c*_ signifies the compressive strength, *F*_*c*_ represents the compressive failure load and *A* denotes the area of the compressed section.

**Fig. 2:**
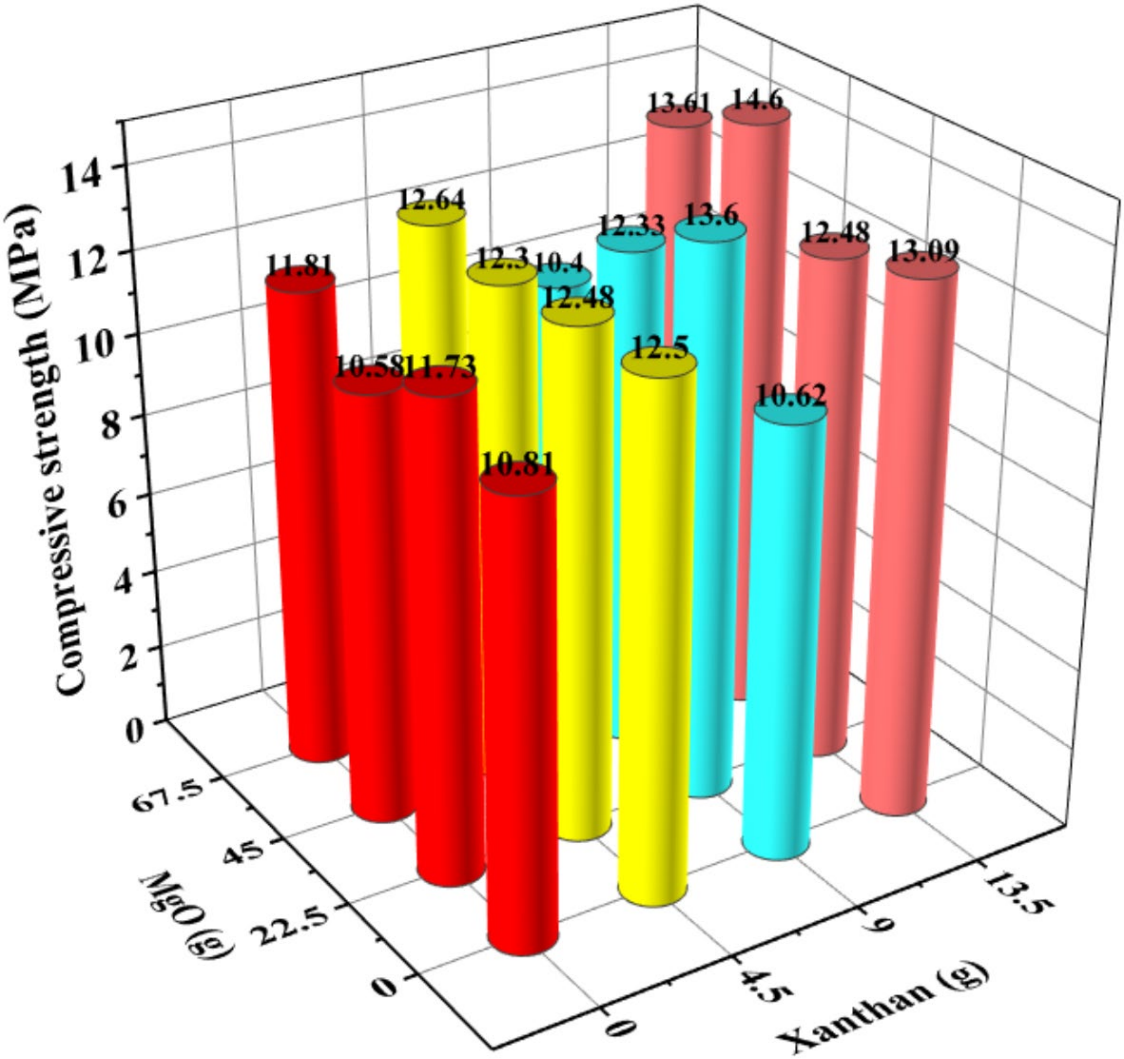
Compressive strength of XG/MgO composite system after 7d.

**Fig. 3:**
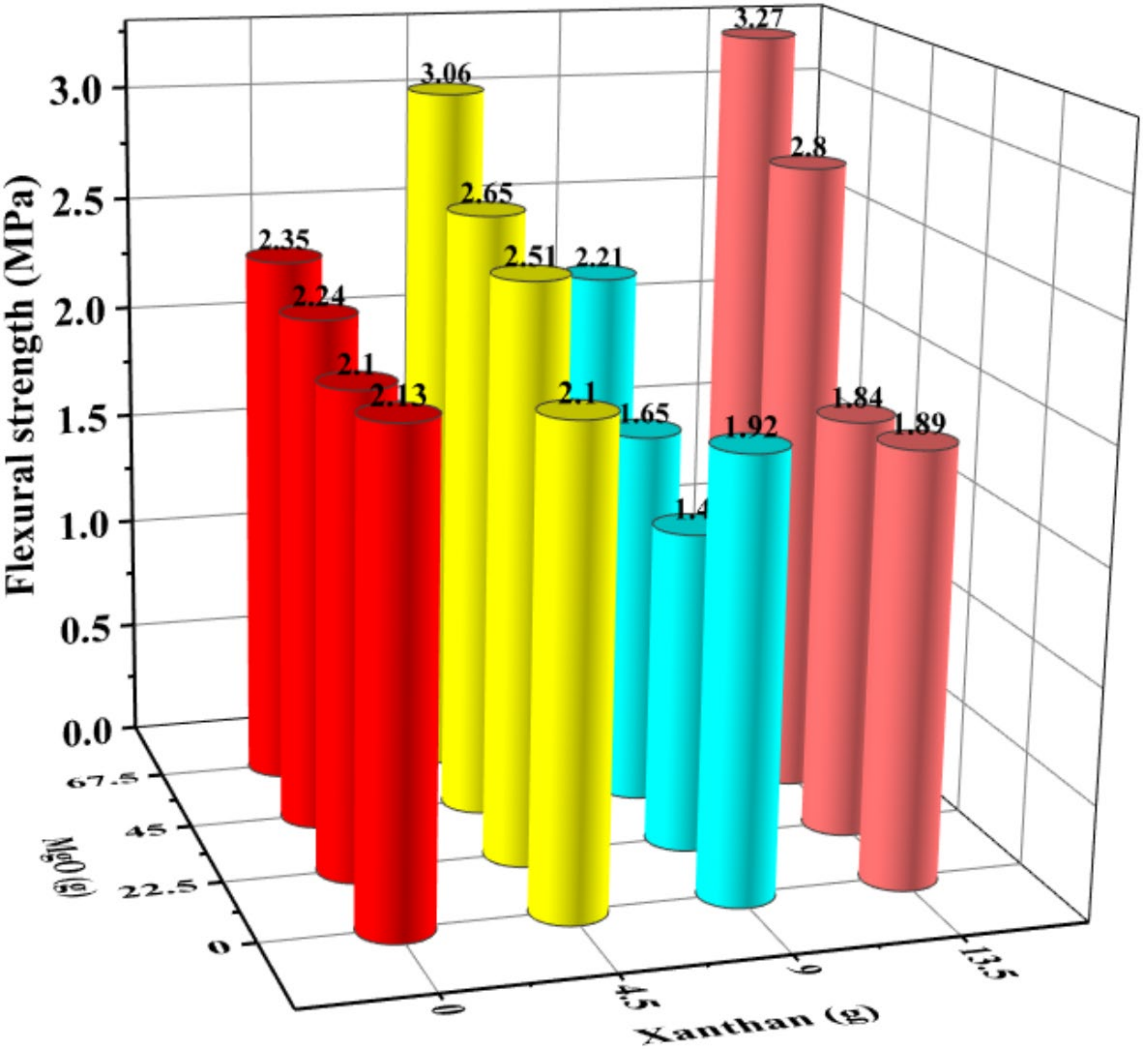
Flexural strength of XG/MgO compound system after 7d.

From Figs. 2 and 3, it can be observed that in the XG/MgO composite system, XG has a significant impact on the compressive strength of CCC, whereas MgO greatly influences its flexural strength. When the MgO content is 5% (45 g) and the XG content is 1.5% (13.5 g), the compressive strength reaches 14.60 MPa, and the flexural strength reaches 2.80 MPa.

Combined with the results of mechanical property analysis, 1.5% XG and 5% MgO were selected as the experimental groups in Table 3, and MWCNTs accounting for 0% (0 g), 0.56% (5 g), 1.11% (10 g), 1.67% (15 g), and 2.22% (20 g) of the mass of cement were added, respectively. This is used to preliminarily determine the reasonable range of MWCNTs, as shown in Table 4.

**Table 4:** Dosage of components in the addition system of MWCNTs.

Serial number	Quality (g)							Water-to-solid ratio
	Cement	Clay	Sodium silicate	Defoamer	MgO	XG	MWCNTs	
1	900	180	15	2	45	13.5	0	0.6
2	900	180	15	2	45	13.5	5	0.6
3	900	180	15	2	45	13.5	10	0.6
4	900	180	15	2	45	13.5	15	0.6
5	900	180	15	2	45	13.5	20	0.6

In order to find out the doping amount of MWCNTs at the optimum mechanical properties of the samples, referring to the literature [22], the dosages of XG, MgO and MWCNTS were redesigned based on Table 4. The design is shown in Table 5.

**Table 5:** Dosage of compoents in the composite system of MWCNTs/XG/MgO.

Serial Number	Quality (g)							Water-to-solid Ratio
	Cement	Clay	Sodium silicate	Defoamer	MgO	XG	MWCNTs	
1	900	180	15	2	45	13.5	7.5	0.6
2	900	180	15	2	45	13.5	12.5	0.6
3	900	180	15	2	45	18	7.5	0.6
4	900	180	15	2	45	18	12.5	0.6
5	900	180	15	2	37.5	13.5	7.5	0.6
6	900	180	15	2	37.5	13.5	12.5	0.6

**Fig. 4:**
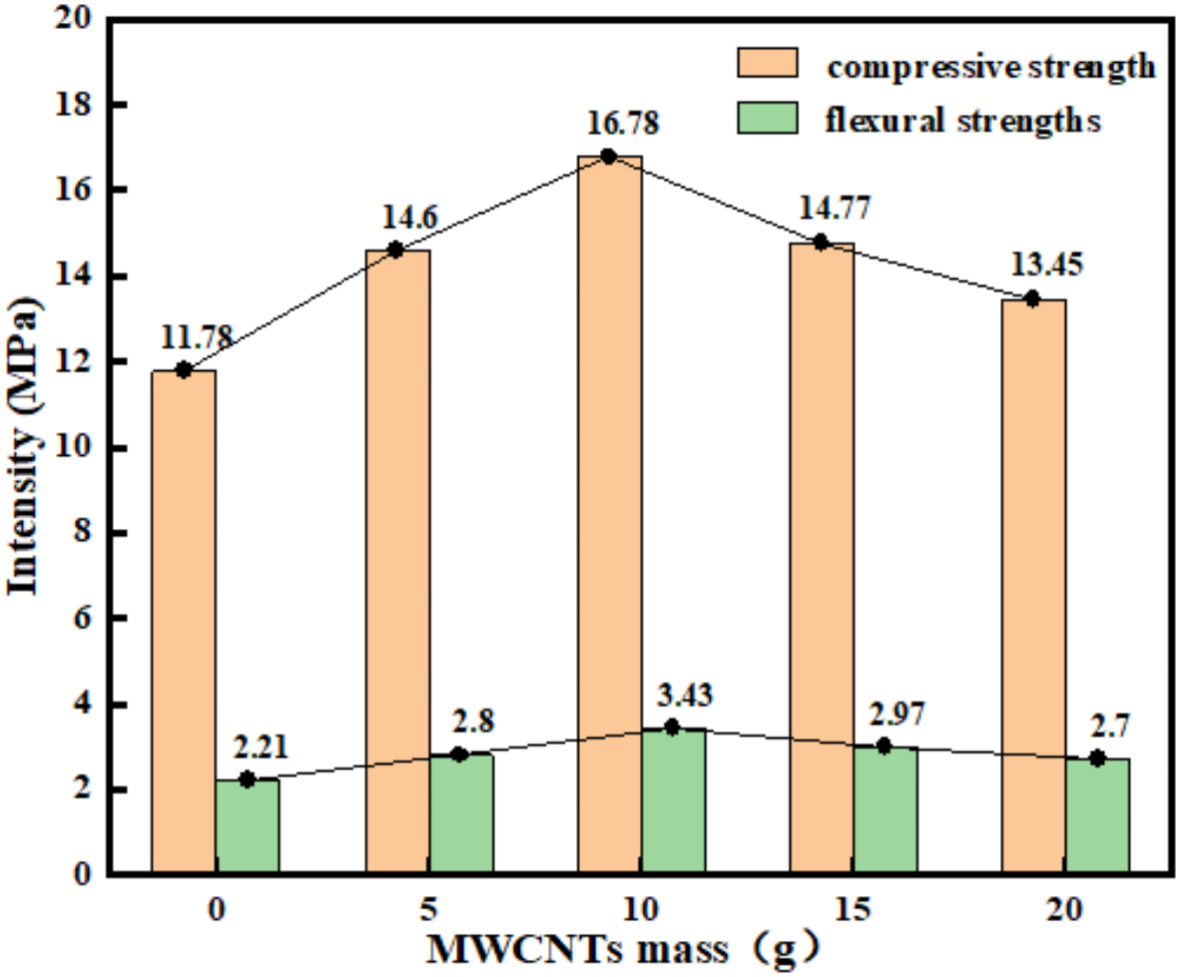
Flexural and compressive strength of the MWCNTs additive system after 7d.

**Fig. 5:**
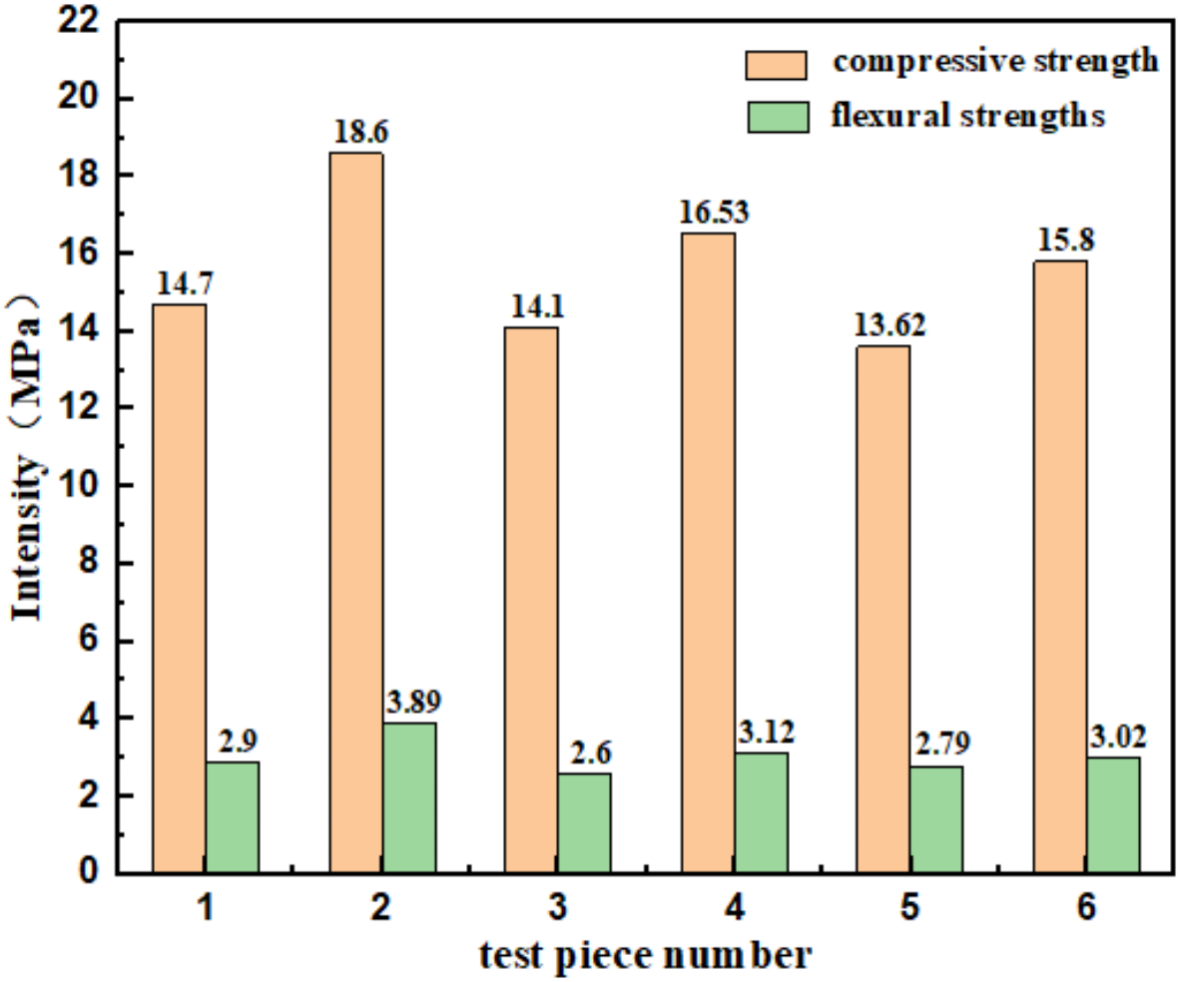
Compressive and flexural strengths of MWCNTs/XG/MgO after 7d.

Figures 4 and 5 indicate that the addition of MWCNTs enhances both the compressive and flexural strengths of the XG-MgO-CCC material. As more MWCNTs are added, the compressive and flexural strengths increase first and then decrease, attributed to excessive MWCNTs causing agglomeration within the material. When the MWCNTs content is 10 g, both strengths are maximized. Consequently, the MWCNTs/XG/MgO composite system is further refined, achieving maximum compressive strength of 18.60 MPa and flexural strength of 3.89 MPa when XG is 13.5 g (1.5%), MgO is 45 g (5%), and MWCNTs are 12.5 g (1.39%).

## Microstructure analysis

This study focuses on the compressive and flexural strengths as well as the pore properties of the materials. The compressive and flexural strengths of the samples with 1.39% of multi-walled carbon nanotubes (MWCNTs) were optimal among the tested samples, so the experimental group with 1.39% of MWCNTs was selected microanalysis (Pore Structure Analysis, XRD, SEM, FTIR) to investigate the micro-mechanism. At the same time, to verify the effect of the addition of MWCNTs, a control group with 0% MWCNTs was selected for simultaneous analysis.

### Pore structure analysis

Based on the mechanical performance analysis results of each sample group, 1.39% MWCNTs from the superior performance group and 0% MWCNTs from the sample group were selected. A liquid nitrogen adsorption test was conducted using specific surface area and pore size analyzers to obtain the pore structure parameters for both sample groups, as shown in Tables 6 and 7.

**Table 6:** Pore structure parameters of 0% MWCNTs.

Sample	Total pore volume	Mean pore size	Porosity	Pore size distribution		
				2–10 nm	10–50 nm	>50 nm
0%MWCNTs	0.21	20.53	19.12	16.89	47.42	35.69

**Table 7:** Pore structure parameters of 1.39% MWCNTs.

Sample	Total pore volume	Mean pore size	Porosity	Pore size distribution		
				2–10 nm	10–50 nm	>50 nm
1.39%MWCNTs	0.19	19.42	17.29	18.20	53.26	28.54

From Tables 6 and 7, it can be observed that in the 0% MWCNTs sample group, the proportion of pore sizes between 2.00 nm and 10.00 nm is 16.89%. In comparison, the proportion of pore sizes between 10.00 nm and 50.00 nm is 47.42%, and the proportion for pore sizes larger than 50.00 nm is 35.69%, resulting in a porosity of 19.12%. In the 1.39% MWCNTs sample group, the proportion of pore sizes between 2.00 nm and 10.00 nm increases to 18.2%, the proportion between 10.00 nm and 50.00 nm rises to 53.26%, and the proportion larger than 50.00 nm decreases to 28.54%, with a porosity of 17.29%. Compared to the 0% MWCNTs sample group, the sample group with added MWCNTs has a lower porosity, a reduced proportion of larger pores, and a more favorable pore size distribution.

### XRD analysis

**Fig. 6:**
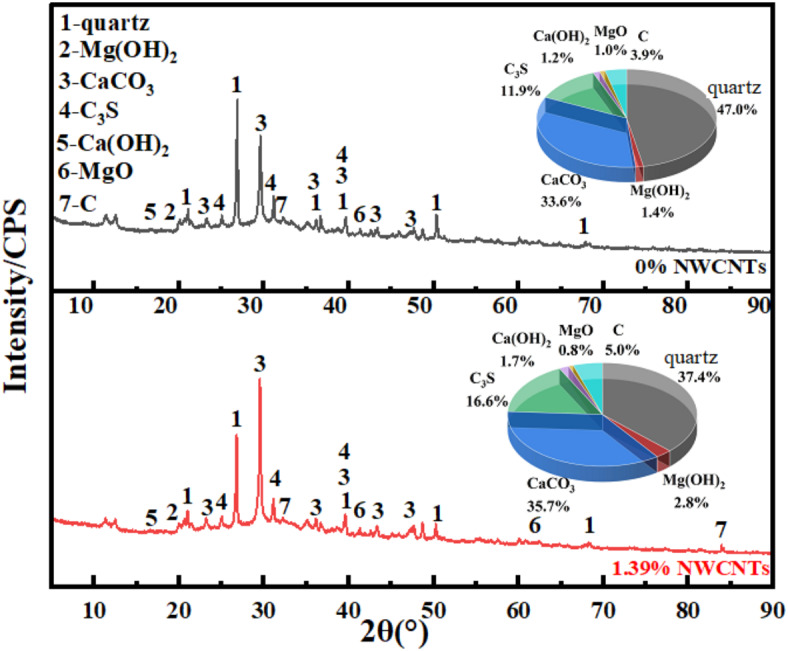
The XRD patterns of the samples with 1.39% MWCNTs and 0% MWCNTs.

X-ray diffraction (XRD) analysis was conducted on the sample group with 1.39% MWCNTs, and the sample group with 0% MWCNTs. The XRD spectra are presented in Fig. 6.

The diffraction peaks of both sample groups clearly correspond to phases including quartz, CaCO_3_, Mg(OH)_2_, and MgO, as shown in Fig. 6. However, compared to the 0% MWCNTs sample group, the diffraction peak of quartz in the 1.39% MWCNTs sample group decreases, while the peak for CaCO_3_ increases and sharpens. It indicates a reduction in the content of quartz and a significant increase in the content of CaCO_3_, which also has a higher crystallinity. The diffraction peaks of MgO and Mg(OH)_2_ are clearly visible in both sample groups, however, the 1.39% MWCNTs sample group contains lower levels of MgO and higher levels of Mg(OH)_2_. Therefore, it can be concluded that incorporating MWCNTs significantly facilitates the reactions between various raw materials, leading to more complete transformations.

### FTIR analysis

**Fig. 7:**
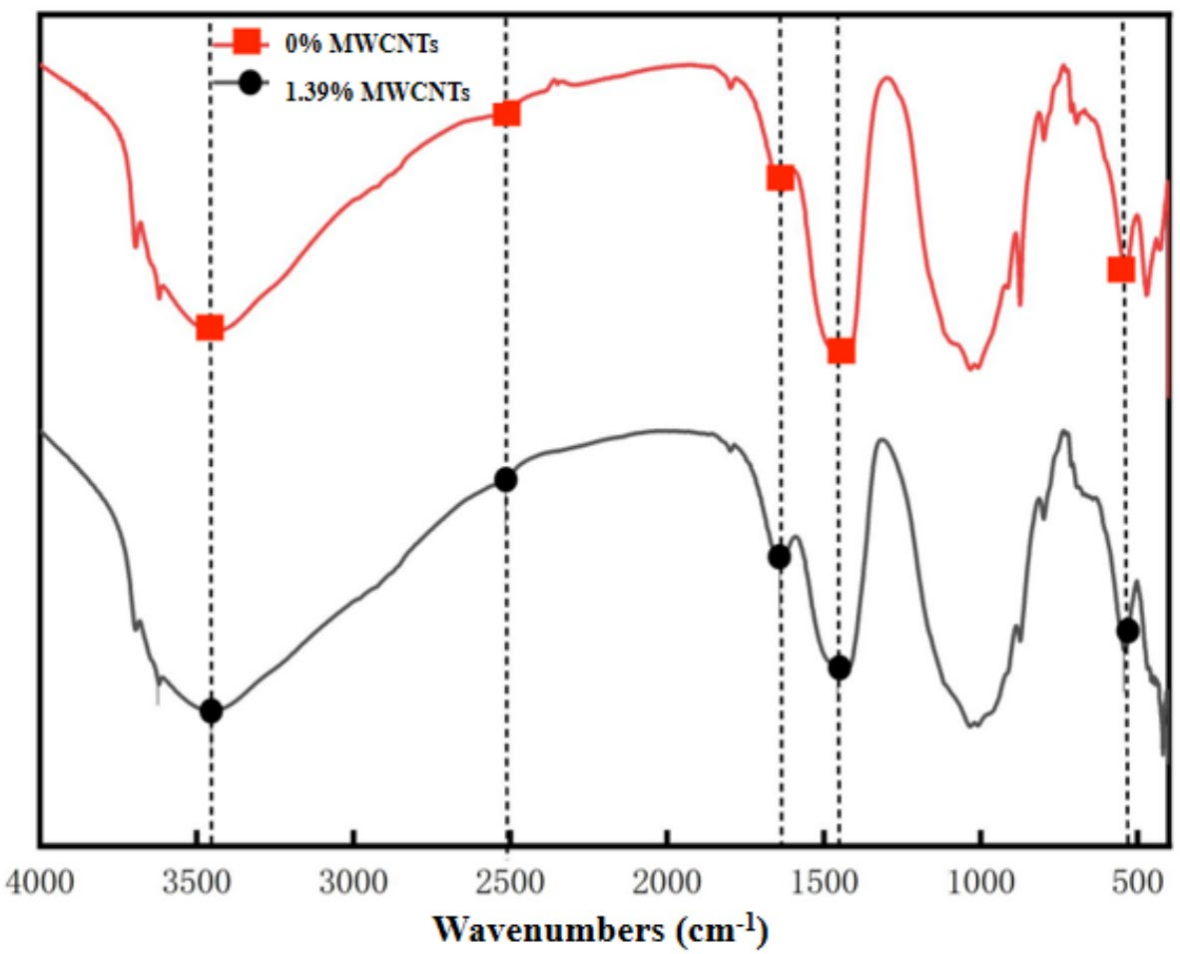
FTIR images of 1.39% MWCNTs and 0% MWCNTs sample groups.

Fourier-transform infrared spectroscopy (FTIR) was used to analyze the mechanical properties of the sample group with 1.39% MWCNTs and the sample group with 0% MWCNTs. The results are shown in Fig. 7.

As shown in Fig. 7, the FTIR spectra of the 0% MWCNTs sample group and the 1.39% MWCNTs sample group do not exhibit significant differences in peak values. Characteristic peaks for carbon compounds were not detected, indicating that MWCNTs did not undergo other chemical reactions that would compromise the structure of the original XG-MgO-CCC material. The absorption peak at 3469 cm^− 1^ corresponds to the stretching vibration of –OH, the peak at 1650 cm^− 1^ is associated with the O–H stretching vibration in H_2_O, the absorption peak at 1456 cm^− 1^ relates to the Ca–O stretching vibration, and the peak at 544 cm^− 1^ represents the Mg–O stretching vibrations in MgO and Mg(OH)_2_. Figure 7 shows that the –OH stretching vibration peak in the 1.39% MWCNTs sample group intensifies while the O–H stretching vibration peak diminishes, alongside a reduction in the Ca–O stretching vibration peak. It suggests that the reaction involving the raw materials in the new material is more thorough, leading to an increased content of products such as Mg(OH)_2_ and ettringite, consistent with the observations made in the XRD analysis.

### Thermogravimetric analysis

**Fig. 8:**
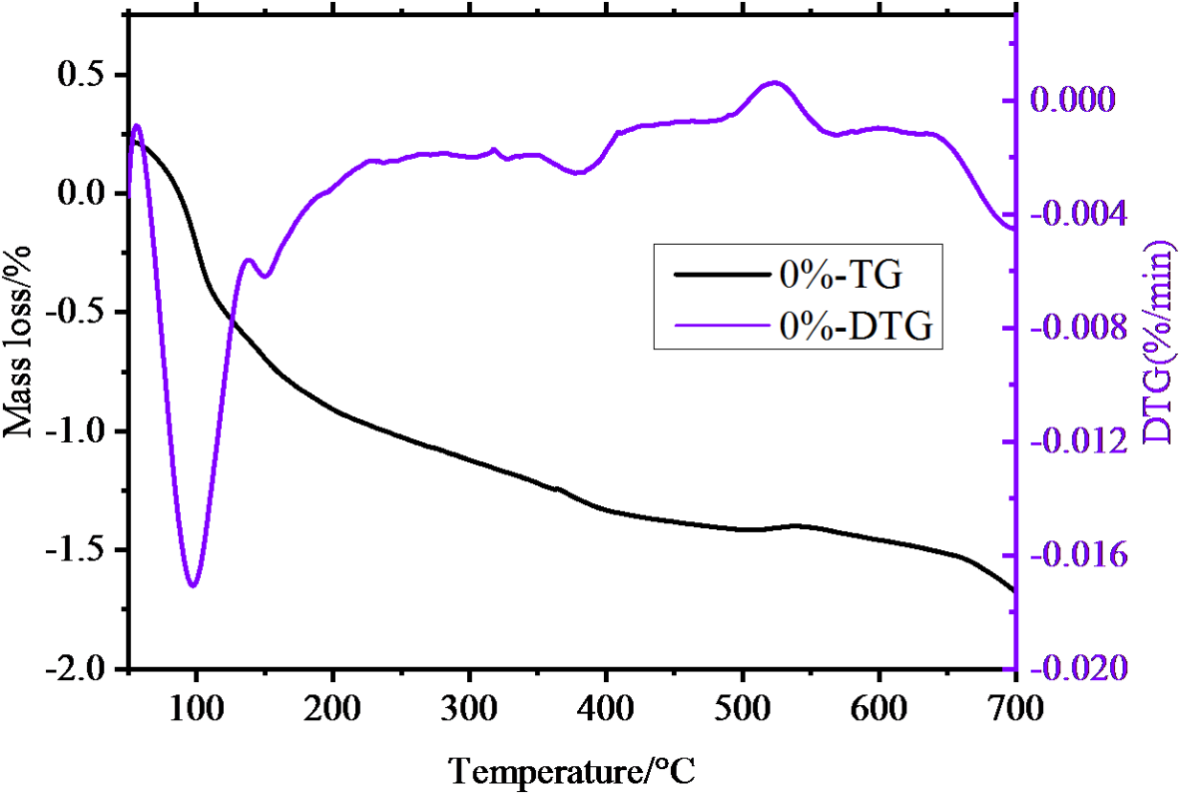
The TG**-DTG** image for the 0% MWCNTs sample group.

**Fig. 9:**
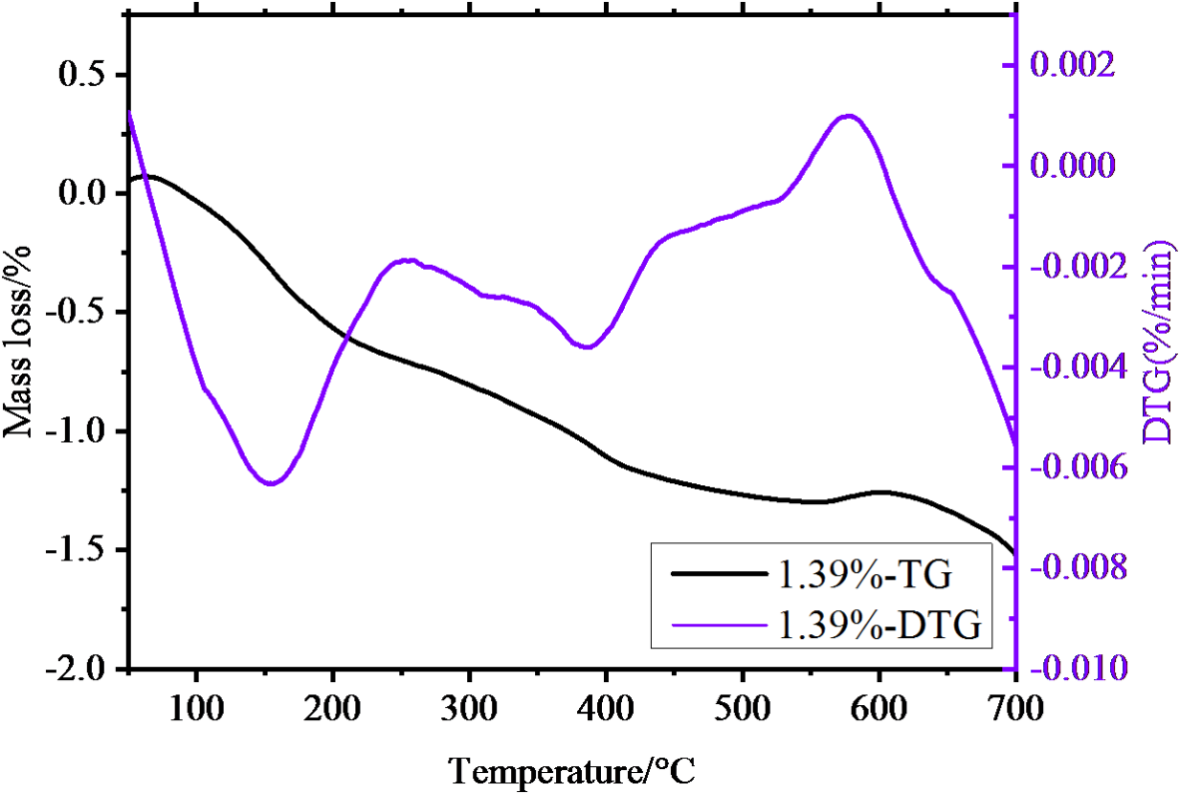
The TG**-DTG** image for the 1.39% MWCNTs sample group.

Thermogravimetric tests were conducted on the 1.39% MWCNTs sample group with excellent mechanical properties and the 0% MWCNTs sample group using a simultaneous thermal analyzer, as illustrated in Figs. 8 and 9.

Both groups of samples underwent continuous mass loss over the temperature range of 0–700 ℃. From Fig. 8, the 0% MWCNTs samples exhibited three primary weight loss ranges. In the TG curve, the first range from 50 to 180 ℃ with a mass loss of 4.24%, a large mass loss occurred in a short period ot time, and the corresponding DTG curve had a sharp peak and a relatively large peak area. The second range of the TG curve was slowly decreasing from 380℃ to 480℃ with a mass loss of 1.08%, and the corresponding DTG curve was relatively flat. The third range was from 500℃ to 700℃ with a mass loss of 1.98%, and the corresponding DTG curve had a small peak. As seen in Fig. 9, the 1.39% MWCNTs samples revealed three major weight loss ranges. A mass loss of 7.33% from 50℃ to 180℃, and the DTG curve had the largest peak and an inflection point, corresponding to the speed of mass loss from fast to slow. The mass loss from 380℃ to 480℃ was 0.53%. The mass loss from 500℃ to 700℃ was 1.77%, showed a peak in the DTG curve, and the rate of the variation was larger than that in the temperature range of 380℃ to 480℃. Considering the weight loss across these ranges, the first range corresponds to the loss of bound water in the hydrated products C-(A)-S-H, the second range primarily involves the decomposition of Ca(OH)2 and Mg(OH)2, while the third range is mainly due to the decomposition of carbonates present in the samples.

### Microstructural analysis

**Fig. 10:**
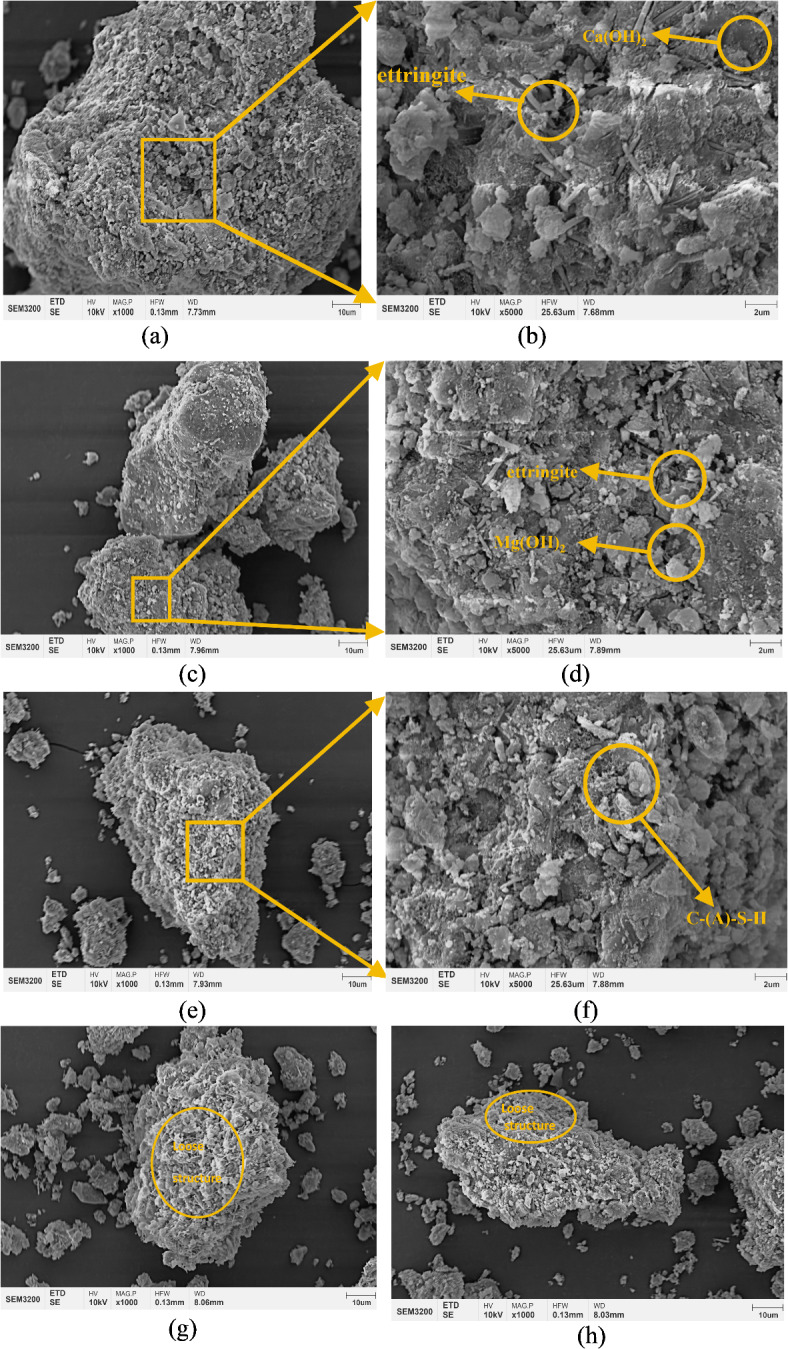
The microstructure of the 1.39% and 0%MWCNTs sample.

Microstructural analysis of the 1.39% MWCNTs sample group was conducted using a scanning electron microscope, as depicted in Fig. 10.

As shown in Fig. 10a and b, layered hydrated calcium oxide and needle-like ettringite can be observed. Figure 10c and d reveal flaky magnesium hydroxide and needle-like ettringite, which provide significant structural support, effectively suppressing the shrinkage of the MXM-CCC and enhancing the stability of the matrix structure. As illustrated in Fig. 10e and f, a network and honeycomb structure of C-(A)-S-H gel can be seen, with a large amount of gel encapsulating substances such as magnesium hydroxide and ettringite. This gel exhibits excellent binding properties, reinforcing the connectivity between materials, resulting in an enhanced pore structure, greater density, and overall integrity of the new material. As shown in Fig. 10g and h, it can be found that compared to the experimental groups, the control group has more pronounced pores in the cementitious materials with more connectivity, resulting in a less dense structure. Therefore, the compressive and flexural strengths of the control group are lower than those of the experimental group.

### Formation mechanism of materials

**Fig. 11:**
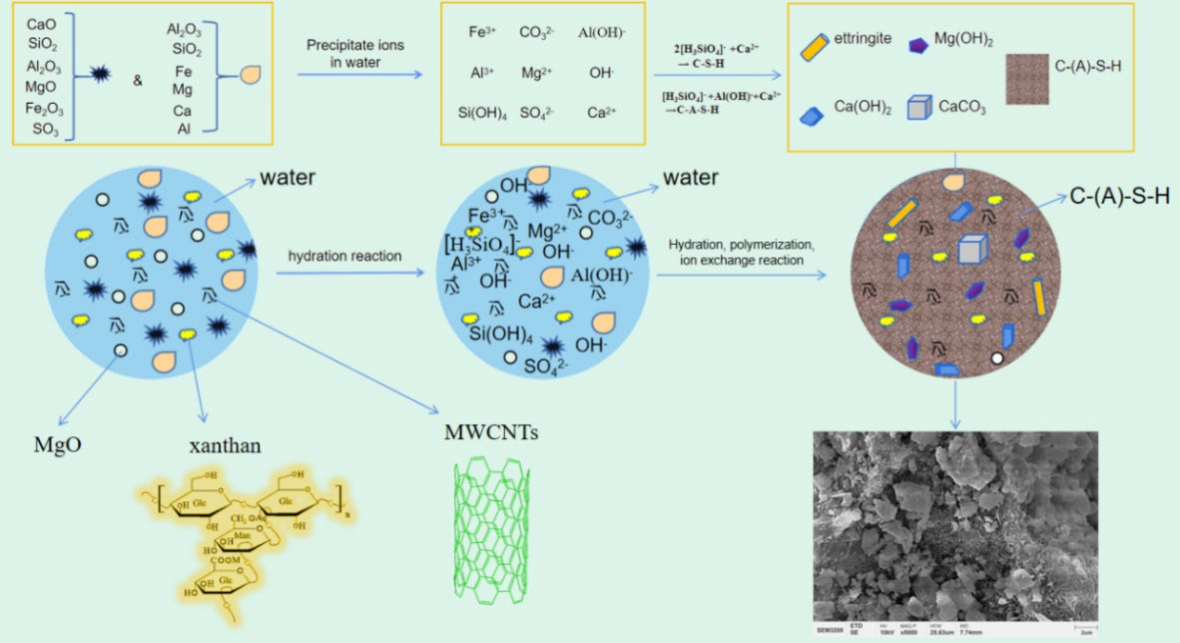
Schematic diagram of the formation mechanism of MWCNTs-XG-MgO-CCC.

The formation mechanism of MXM-CCC is illustrated in Fig. 11. Combined with the results of XRD, FTIR, SEM and TG-DTG analysis, it is known that the raw materials contained CaCO3, Mg(OH)2, and MgO. After the hydration reaction started, MgO and Ca-containing substances in the MXM-CCC complex group underwent a hydrolysis reaction in the reaction system to form Mg2+, Ca2+. The remaining raw materials formed various ingredients such as -OH, -COOH, etc^22^. After the beginning of the hydration reaction, the products of gel C-S-H, C-A-S-H gradually increased. In particular, the addition of MWCNTs further promoted the hydration reaction, the pore size decreased significantly, the pore connectivity weakened, and the microstructure denser, which increased the compressive and flexural strength of the samples to some extent^30–33^. Through the mass loss in the three temperature ranges of the TG-DTG curves, it can also be revealed that the addition of MWCNTs, which promotes the hydration reaction, increased the gel material and significantly improved the mechanical properties^34,35^.

## Conclusion

In this paper, MWCNTs, supplemented by MgO and XG, were utilized to modify CCC. Based on the experimental results, the following conclusions can be made:

1. Analysis of the compressive and flexural strengths of various sample groups shows that XG has a significant effect on the compressive strength of the material, while MgO provides a certain enhancement to the flexural strength. When the MgO content is 5%, and the XG content is 1.5%, the compressive strength reaches 14.60 MPa, and the flexural strength reaches 2.80 MPa. The addition of MWCNTs to the XG-MgO-CCC material significantly improves both compressive and flexural strengths. When the MWCNTs content is 1.39%, the 7-day compressive strength of MXM-CCC reaches 18.60 MPa, and the flexural strength reaches 3.89 MPa.
2. Combining pore structure and micromorphology analysis, the proportion of pore sizes in MXM-CCC within the range of 2.00 nm to 10.00 nm is 18.2%, while that of pore sizes between 10.00 nm and 50.00 nm is 53.26%, and for pore sizes greater than 50.00 nm, it accounts for 28.54%. The porosity is measured at 17.29%. According to thermogravimetric analysis, the new material shows an increased formation of ettringite and calcium silicate hydrate, with the addition of MWCNTs significantly enhancing the hydration reaction of the material, resulting in a more thorough reaction.
3. The characterization results of MXM-CCC indicate that the incorporation of MWCNTs facilitates the progress of the hydration reaction, acting as a “bridge” that connects hydration products and performs its role as a nano-filler, further optimizing the pore size of MXM-CCC. The addition of XG encourages the formation of dense chelates of Ca^2+^ within MXM-CCC, filling in some of the larger pores and connecting clay surface particles with hydration products. The hydration reaction of MgO produces Mg(OH)_2_ crystals that fill the pores within the material. Consequently, MXM-CCC exhibits reduced porosity and a more excellent pore size distribution, resulting in a denser material structure and superior mechanical properties.

## Data Availability

All data generated or analysed during this study are included in this published article.
